# The frequency of osteolytic bone metastasis is determined by conditions of the soil, not the number of seeds; evidence from in vivo models of breast and prostate cancer

**DOI:** 10.1186/s13046-015-0240-8

**Published:** 2015-10-20

**Authors:** Ning Wang, Kimberley J. Reeves, Hannah K. Brown, Anne C M Fowles, Freyja E. Docherty, Penelope D. Ottewell, Peter I. Croucher, Ingunn Holen, Colby L. Eaton

**Affiliations:** The Mellanby Centre for Bone Research, Department of Human Metabolism, Medical School, University of Sheffield, Beech Hill Road, Sheffield, S10 2RX UK; Department of Oncology, Medical School, University of Sheffield, Sheffield, UK; Break Through Breast Cancer Research Unit, Paterson Institute for Cancer Research Manchester, Manchester, UK; Bone Biology Division, Garvan Institute of Medical Research, Sydney, Australia

**Keywords:** Tumor-induced bone disease, Bone remodelling, Two-photon microscopy, Animal models

## Abstract

**Background:**

While both preclinical and clinical studies suggest that the frequency of growing skeletal metastases is elevated in individuals with higher bone turnover, it is unclear whether this is a result of increased numbers of tumour cells arriving in active sites or of higher numbers of tumour cells being induced to divide by the bone micro-environment. Here we have investigated how the differences in bone turnover affect seeding of tumour cells and/or development of overt osteolytic bone metastasis using *in vivo* models of hormone-independent breast and prostate cancer.

**Methods:**

Cohorts of 6 (young) and 16 (mature)-week old BALB/c nude mice were culled 1, 7 and 21 days after received intracardiac injection of luciferase expressing human prostate (PC3) or breast cancer (MDA-MB-231) cell lines labelled with a fluorescent cell membrane dye (Vybrant DiD). The presence of growing bone metastases was determined by bioluminescence using an in vivo imaging system (IVIS) and followed by anatomical confirmation of tumour metastatic sites post mortem, while the presence of individual fluorescently labelled tumour cells was evaluated using two-photon microscopy *ex vivo*. The bone remodelling activities were compared between young and mature naïve mice (both male and female) using micro-CT analysis, ELISA and bone histomorphometry.

**Results:**

Both prostate and breast cancer cells generated higher numbers of overt skeletal lesions in young mice (~80%) than in mature mice (~20%). Although mature mice presented with fewer overt bone metastases, the number of tumour cells arriving/colonizing in the tibias was comparable between young and mature animals. Young naïve mice had lower bone volume but higher bone formation and resorption activities compared to mature animals.

**Conclusions:**

Our studies suggest that higher frequencies of growing osteolytic skeletal metastases in these models are linked to increased bone turnover and not to the initial number of tumour cells entering the bone microenvironment.

## Background

About 90% patients with advanced breast and prostate cancer have incurable bone metastases [1, 2], with a mean survival of one year [3, 4]. Treatments that aim to either prevent or suppress the growth of bone metastasis are limited. Anti-bone resorptive agents such as zoledronic acid, prevent bone loss and skeletal-related events (SREs), but do not increase survival in unselected patients with advanced disease [4–7]. However, a better understanding of the early mechanisms leading to the development of bone metastasis would allow such therapies to be specifically targeted to early lesions as they arise.

The ‘seed and soil hypothesis’ for metastasis proposes that only the right cells disseminated from primary tumour (the ‘seeds’) landing in the right microenvironment (the ‘soil’) can form secondary (metastatic) tumours successfully [8]. It has been suggested that prostate and breast cancers contain a small percentage (<1%) of stem cell-like populations and that it is these cells that have the potential to act as the ‘seeds’, leaving the primary site, surviving in the circulation, homing into the bone, where they eventually form bone metastases [9–14]. In this context, bone is the fertile ‘soil’, providing a supportive microenvironment (‘bone metastasis niche’) for tumour cell survival and secondly, under the right conditions, for the proliferation of clinically relevant metastases. New evidence has shown that disseminated prostate cancer cells target the haemopoietic stem cell niche to establish footholds in bone, with the osteoblastic lineages as the key components of the niche [15, 16]. In addition, bone continuously renews itself with precisely balanced osteoblastic bone formation and osteoclastic bone resorption. Elevated levels of bone turnover have been shown to be correlate with increased numbers of metastases, in various xenograft models and clinical studies [17–22]. Whether this effect is a result of different bone environments increasing tumour cell seeding, or of environments inducing proliferation of resident tumour cells in bone to form overt metastases, remains to be determined.

In this study, we investigated the initiation of prostate and breast cancer bone metastases in young (6-week old) and in mature (16-week old) athymic mice. Both bone turnover rates and the frequency of metastases were lower in the mature compared to the younger animals allowing us to study the initiation of metastases in different bone microenvironments. We used two-photon microscopy to identify initial seeding of tumour cells into bone and compared this with the frequency of growing metastases. This is the first study to directly test the hypothesis that the frequency of bone metastasis is equally dependent on numbers of seeded tumour cells and growth induction by the bone microenvironment.

## Methods

### Mice

All studies were performed using 6-week or 16-week old BALB/cAnNCrl immunocompromised (athymic nude) mice (Charles River, Kent, UK) as xenograft models of bone metastasis. All procedures complied with the UK Animals (Scientific Procedures) Act 1986 and were reviewed and approved by the local Research Ethics Committees of the University of Sheffield under Home Office project licence 40/3462 (Sheffield, UK).

### Cell lines

The human prostate cancer cell line PC3 (ATCC, Middlesex, UK) and human breast cancer cell line MDA-MB-231 (ATCC, Middlesex, UK) were stably transfected with a firefly luciferase gene luc2 (pGL4.51 [luc2/CMV/Neo] vector, Promega, Southampton, UK) and denoted as PC3-NW1 and MDA-MB-231 luc2. Both cell lines were maintained in Dulbecco’s Modified Eagle Medium (DMEM) (Life Technologies, Paisley, UK), supplemented with 100 Units/mL Penicillin, 100 μg/mL Streptomycin and 10% foetal calf serum (FCS) (Sigma Aldrich Co Ltd, Poole, UK).

### Xenograft models

Human cancer cells were initially stained with 5 μM lipophilic carbocyanine dye Vybrant DiD (Life Technologies, Paisley, UK) according to the manufacture’s protocol. Using the Vybrant DiD dye, which is lost as cells divide, allows non-dividing/slowly dividing cells to be identified and distinguished from proliferating cells [15, 23, 24]. A single-cell suspension of 1 × 10^5^ DiD labelled PC3-NW1 cells/100μL PBS and 0.75x10^5^ DiD labelled MDA-MB-231 luc2 cells/100μL PBS were injected into the left cardiac ventricle (intracardiac (i.c.) injection) of 6- or 16-week old male and female BALB/c nude mice, respectively. Overt tumours were monitored up to 8 weeks post injection using an in vivo imaging system (IVIS, PerkinElmer, Cambridge, UK) and the tumour burden was measured based on radiance of luminescence using the Living Image software (PerkinElmer), followed by anatomical confirmation of tumour metastatic sites post mortem. Cohorts of animals (minimum *n* = 6/group) were euthanized on day 1, 7 and 21 post injection and individual non-dividing DiD labelled tumour cells were quantified in the tibiae *ex vivo* by two-photon microscopy, to understand the quantity of tumour cells arriving and colonizing within the bone marrow.

### Two-photon microscopy

Dissected right tibiae were prepared and sectioned as described previously [15, 24]. An area of 2104 μm × 2525 μm below the growth plate with 100 μm in depth was imaged using a Zeiss LSM510 NLO upright multiphoton/confocal microscope (Carl Zeiss Inl, Cambridge, UK). A 633nm HeNe laser was used to detect DiD labelled cells while the bone was detected using the 900nm Chameleon multiphoton laser. The number of tumour cells (DiD positive events) and their distance to the nearest bone surface (a parameter indicating the location of tumour cells towards osteoblastic niche) was analysed using the Volocity 3D Image Analysis software 6.01 (PerkinElmer, Cambridge, UK).

### Micro-CT analysis

Right femurs were dissected and scanned by SkyScan 1172 desktop micro-CT (SkyScan) at the resolution of 6 μm. Trabecular morphometry, characterized as trabecular bone content (BV/TV) was measured from a 1.0mm thick region 0.2mm above the growth plate where metastatic tumour cells are generally situated. Nomenclature and symbols were used to describe the micro-CT derived bone morphometries according to the published guidelines [25].

### Serum bone remodelling markers

Tartrate-resistant acid phosphatase 5b (TRAP) activity in mouse serum was determined as a measure of bone resorption activity using an IDS MouseTRAP™ Assay (Immunodiagnostic Systems, Tyne & Wear, UK). Type 1 procollagen amino-terminal-propeptide (P1NP) and osteocalcin were determined for bone formation activities, using a Rat/Mouse P1NP competitive immunoassay kit (Immunodiagnostic Systems, Tyne & Wear, UK) and a Mouse Osteocalcin ELISA Kits (Takara Bio Europe, Saint Germain, France), for male and female mice samples, respectively.

### Bone histomorphometry

Dissected left tibiae were prepared and TRAP stained as described previously [26]. The number of osteoblasts (N.Ob/B.Pm), the bone surface covered by osteoblasts (Ob.Pm/B.Pm), the number of osteoclasts (N.Oc/B.Pm), and the bone surface covered by osteoclasts (Oc.Pm/B.Pm) were determined on a 1.5mm length of endocortical surfaces, using a DMRB microscope (Leica Microsystems, Wetzlar, Germany) with the Osteomeasure bone histomorphometry software (OsteoMetrics, Inc. Decatur, GA, USA).

### Statistic analysis

All data are expressed as mean ± SEM. Statistical significance was tested for using an unpaired Student’s t-test using the Prism 6 software (GraphPad, La Jolla, USA). *P* < 0.05 was considered to be significant.

## Results

### The frequency of overt, skeletal prostate cancer metastases was elevated in young compare to mature male mice

**Fig. 1:**
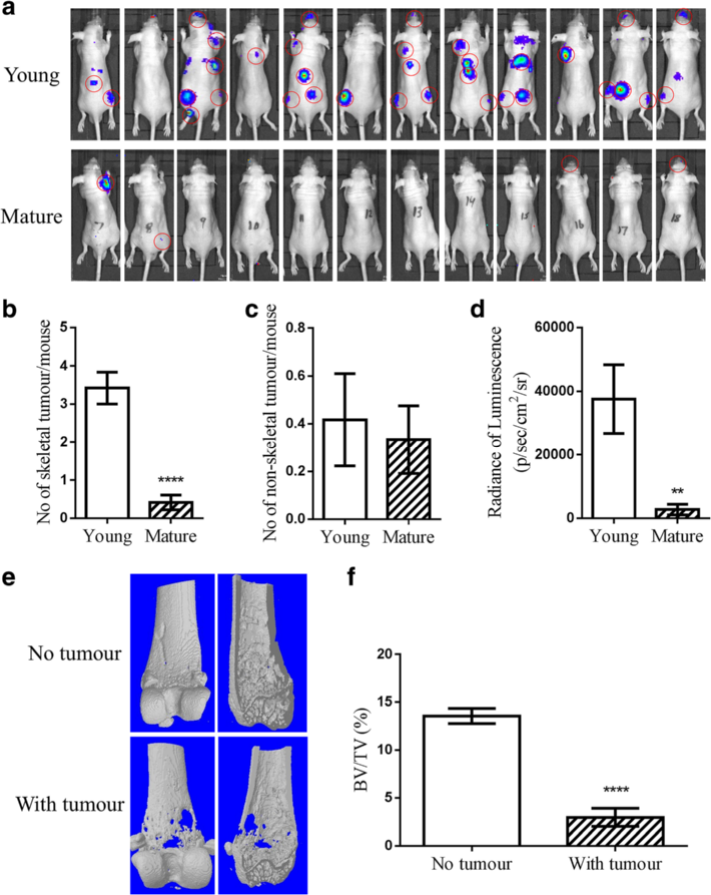
Comparison of skeletal metastases by human prostate cancer cells in young and mature male mice. A single-cell suspension of 1 × 10^5^ DiD labelled PC3-NW1 cells/100 μL PBS was injected into the left cardiac ventricle of 6-week old (Young) or 16-week old (Mature) male BALB/c nude mice. **a** Tumour growth was monitored by in vivo imaging. Skeletal tumours (red circled) were identified and confirmed by further anatomical examination post mortem up to 8 weeks post injection. **b** The number of skeletal tumours per mouse, **c** the number of non-skeletal tumour per mouse (skeletal muscle, liver, adrenal glands, but not peri- or intra-cardial tumours), and **d** the total tumour burden measured by radiance of luminescence were also compared between young and mature mice, *n* = 12. **e** A 3D bone models created using the micro-CT scan on tumour bearing femurs in young mice shows the bone lesions caused by these tumours. **f** To measure the bone destruction in trabecular compartment, trabecular bone content (BV/TV) were then quantified and compared between tumour bearing and non-tumour bearing legs of young mice, *n* > 8. (** *P* < 0.01, ****P < 0.0001, t-test)

We first established how prostate cancer cells formed tumours in bone following intra-cardiac injection, comparing young and mature male mice. Three weeks after tumour cell injection, young animals developed tumours, observed by bioluminescence, with an overall skeletal tumour frequency of greater than 80% in multiple experiments (*n* = 12 in total). In contrast, only 20% of mature animals developed skeletal tumours (*n* = 12) three weeks post injection and 40% of mature animals developed skeletal tumours by eight weeks (Fig. 1a). The young mice had significantly higher number of skeletal tumours (3.4 ± 0.4 tumours per mice vs 0.4 ± 0.2, *p* < 0.0001), but not non-skeletal tumours in soft tissue and organs, such as in skeletal muscle, liver, adrenal glands, except peri- or intra-cardial tumours caused by incorrect injections, compared to mature mice (0.4 ± 0.2 vs 0.3 ± 0.1, *p* = 0.7314)(Fig. 1b and c). This was reflected by the total tumour burden determined by the radiance of tumour luminescence [37556 ± 10797 vs 2754 ± 1689 photons/sec/cm^2^/steradian (p/sec/cm^2^/sr), *p* = 0.0043] (Fig. 1d). The skeletal tumours clearly caused osteolytic bone lesions in both young and old mice, as shown in the 3D micro-CT scanning image of a tumour bearing femur of young mice associated with bone destruction (Fig. 1e). This was confirmed by significant decrease in trabecular bone content (BV/TV) compared to non-tumour bearing femurs (13.6 ± 0.8 vs 3.0 ± 0.9, *p* < 0.0001)(Fig. 1f).

### Seeding of prostate cancer cells to bone was not increased in young mice

**Fig. 2:**
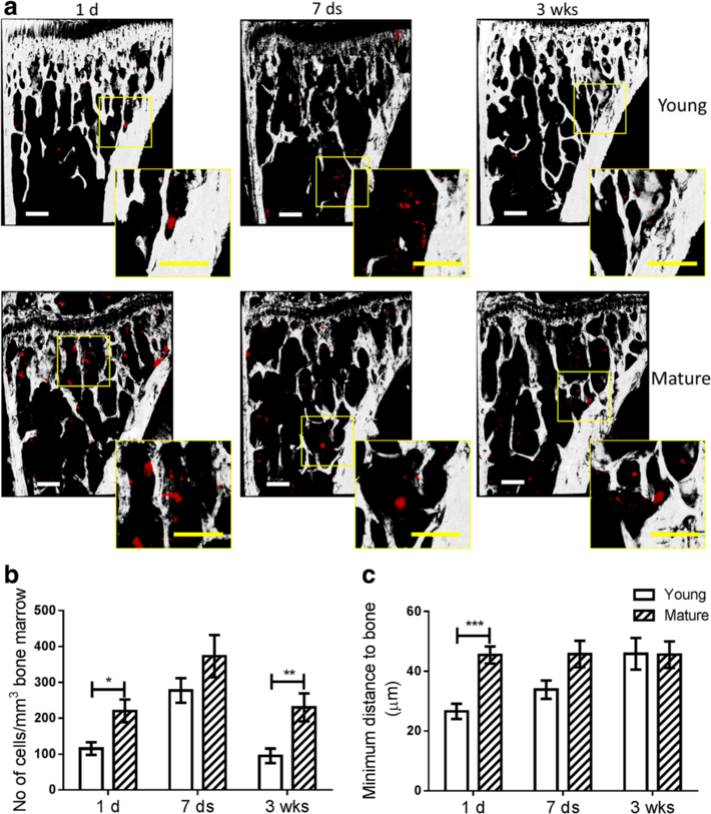
Quantification of human prostate cancer cells arriving and colonizing the bone marrow of male mice. **a** The DiD labelled tumour cells entering the bone microenvironment were visualized and quantified using two-photon microscopy on day 1, 7 and 21 post tumour cells injection. Scale bar = 200μm. **b** The number of tumour cells per mm^3^ bone marrow volume and **c** the minimum distance from tumour cells to the nearest bone surface was quantified and compared between 6-week old (Young) or 16-week old (Mature) mice using the Volocity 3D Image Analysis software. *n* > 6, * *P* < 0.05, ** *P* < 0.01, *** *P* < 0.001, t-test

The higher frequency of tumours in bone could be due to increased numbers of tumour cells seeding in the niche in young animals. We therefore quantified the number of DiD-labelled prostate cancer cells in the tibia of male mice using two-photon microscopy *ex vivo* and found that increased tumour cell seeding was not responsible for this higher frequency of bone metastases observed in younger animals. DiD labelled prostate cancer cells were identified in tibial bone marrow of both younger and older mice at different time points (1 day, 7 days, and 3 weeks) following intra cardiac injection (Fig. 2a). Quantified using Volocity 3D image analysis software, the time course of tumour cells homing to the bone marrow showed a very similar pattern in younger and older mice, with the number of resident tumour cells reaching its peak 7 days post-injection. Significantly fewer tumour cells were present in younger mice on day 1 and at 3 weeks post-injection (Fig. 2b). Fewer cells were also observed in younger mice on day 7 but this failed to reach significance. Tumour cells were found located in close proximity (within a 50 μm range) to bone at all time-points in both age groups, but were found to be located significantly closer to bone in young animals than in mature animals 1 day post-injection. This trend continued on day 7 but was not present at 3 weeks (Fig. 2c).

### Bone turnover was higher in young compared to mature mice

**Fig. 3:**
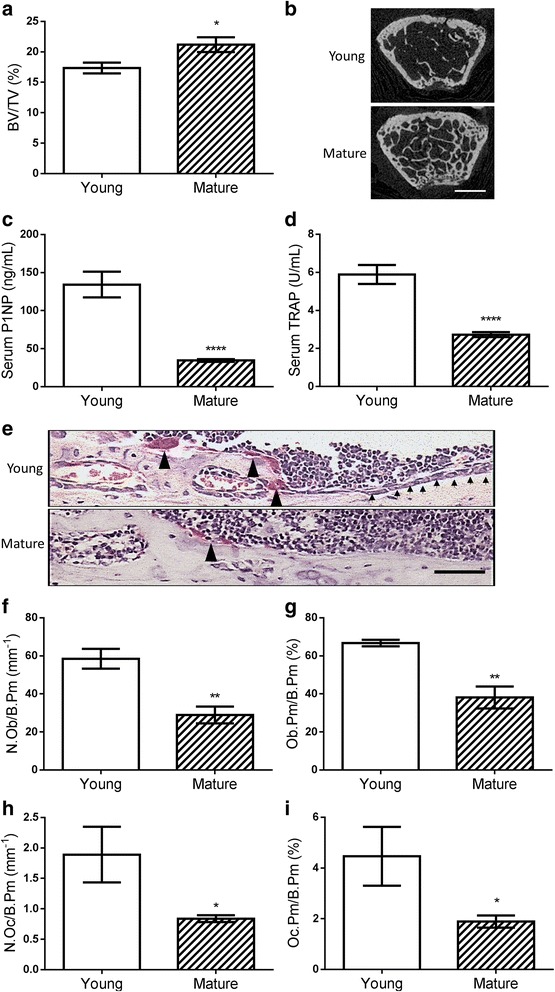
Differences of bone remodelling activities between younger and older male BALB/c nude mice. **a** The trabecular bone content (BV/TV) was compared between 6-week old (Young) or 16-week old (Mature) male mice using micro-CT analysis. *n* > 9. **b** The trabecular bone mass difference can be clearly seen on the cross-section micro-CT image 250 μm below the growth plate, scale bar = 500μm. **c** The bone formation marker (P1NP) and **d** bone resorption marker (TRAP) were also measured in mouse serum sample using ELISA kits, *n* > 9. **e** The bone resorbing osteoclasts (TRAP staining +, marked by black arrow heads) and bone forming osteoblasts (marked by small black arrows) were viewed and quantified on the endocortical bone surface of 6- and 16-week old mice tibiae after sectioning and TRAP staining. Scale bar = 50μm. **f** The number of osteoblasts per mm endocortical bone surface (N. Ob/B.Pm), **g** bone surface covered by osteoblasts (Ob.Pm/B.Pm), **h** number of osteoclasts per mm endocortical bone surface (N.Oc/B.Pm), and **i** bone surface covered by osteoclasts (Oc.Pm/B.Pm) were all compared between 6- and 16-week old mice. *n* > 4, * *P* < 0.05, ** *P* < 0.01, **** *P* < 0.0001, t-test

As the number of tumour cells seeded into bone is unlikely to account for the increase in overt metastases in young animals, we explored the effects of changes in the bone microenvironment to this process. Using naïve mice, we carried out detailed micro-CT analysis on distal regions of femurs and showed that the young male mice had 18% lower BV/TV compared to mature male mice (17.4 ± 0.9 vs 21.2 ± 1.2, *p* = 0.0194)(Fig. 3a and b). However, the younger mice have greater overall bone remodelling activities than older mice, with evidence of nearly 3 fold more bone formation (134.3 ± 17.0 vs 34.5 ± 1.7 ng/mL, *p* = 0.0004) and increased bone resorption activities (5.9 ± 0.5 vs 2.7 ± 0.1 U/mL, *p* = 0.0002), determined via quantifying serum P1NP and TRAP concentrations, respectively (Fig. 3c and d). This is consistent with histomorphometric analysis of the osteoblasts and TRAP positive osteoclasts on the endocortical bone surface of the tibiae (Fig. 3e). These data showed that young male mice had increased numbers of osteoblasts (58.5 ± 0.5 vs 28.9 ± 4.4 mm^−1^, *p* = 0.0024)(Fig. 3f), increased areas of osteoblast covered bone surfaces (66.7 ± 1.7 vs 38.2 ± 5.8 %, *p* = 0.0056) (Fig. 3g), increased numbers of osteoclasts (1.8 ± 0.5 vs 0.8 ± 0.1 mm^−1^, *p* = 0.0127)(Fig. 3h), and 2 fold more osteoclast covered bone surfaces (4.5 ± 1.2 vs 1.5 ± 0.2 %, *p* = 0.0187)(Fig. 3i) than mature animals.

In order to ensure that the above results were not unique to the prostate cancer model we repeated the studies using breast cancer cells injected in female mice, assessing tumour cell seeding in bone, progression of metastases and bone turnover.

### The frequency of overt, skeletal breast cancer metastases was elevated in young compared to mature female mice

**Fig. 4:**
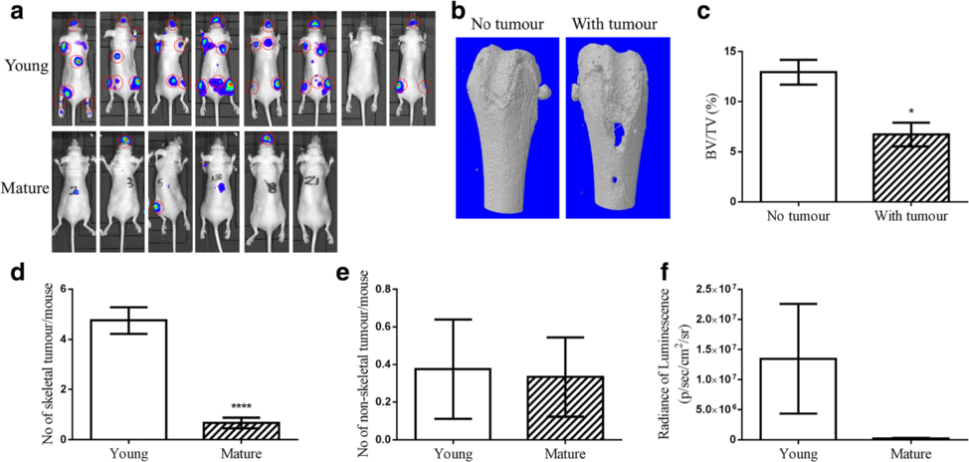
Comparison of skeletal metastases by human breast cancer cells in young and mature female mice. A single-cell suspension of 0.75 × 10^5^ DiD labelled MDA-MB-231-luc2 cells/100 μL PBS was injected into the left cardiac ventricle of 6-week old (Young) or 16-week old (Mature) female BALB/c nude mice. **a** Tumour growth was monitored using the in vivo imaging system (IVIS). Skeletal tumours (red circled) were identified and confirmed by further anatomical examination post mortem up to 8 weeks post injection. **b** The bone lesions were examined and confirmed from 3D bone models created via micro-CT scanning tumour bearing femurs of young mice. **c** BV/TV was then quantified and compared between tumour bearing and non-tumour bearing legs of young mice, *n* = 4. **d** The number of skeletal tumours per mouse, **e** the number of non-skeletal tumour per mouse (skeletal muscle, liver, adrenal glands, but not peri- or intra-cardial tumours), and **f** the total tumour burden (radiance of luminescence) were also compared between 6- and 16-week old mice, *n* > 6. (* *P* < 0.05, ****P < 0.0001, t-test)

Three weeks after injection of DiD-labelled MDA-MB-231 human breast cancer cells, young female mice had developed significantly higher numbers of tumours in the skeleton (~80%) compared to mature animals (~50% skeletal tumours at both 3 and 8 weeks post injection) (Fig. 4a). The tumours caused lytic bone lesions in both young and mature mice, as shown in the 3D micro-CT images of tumour bearing femurs of young mice (Fig. 4b) and led to significant decrease in BV/TV (12.9 ± 1.2 vs 6.7 ± 1.7, *p* = 0.0107) (Fig. 4c). Younger female mice carried an average of 4.8 ± 0.5 skeletal tumours compared to 0.7 ± 0.2 in older mice (*p* < 0.0001)(Fig. 4d). There was no difference in non-skeletal tumour frequency (skeletal muscle, liver, brain but not peri- or intra-cardial tumours) between the groups (0.4 ± 0.2 vs 0.3 ± 0.2, *p* = 0.9087)(Fig. 4e). The skeletal tumour burden in younger female mice was about 56 fold higher than that of older mice (13470000 ± 9121000 vs 233787 ± 82546 p/sec/cm2/sr, *p* = 0.2373)(Fig. 4f).

### Similar numbers of breast cancer cells were present in bone of young and mature female mice

**Fig. 5:**
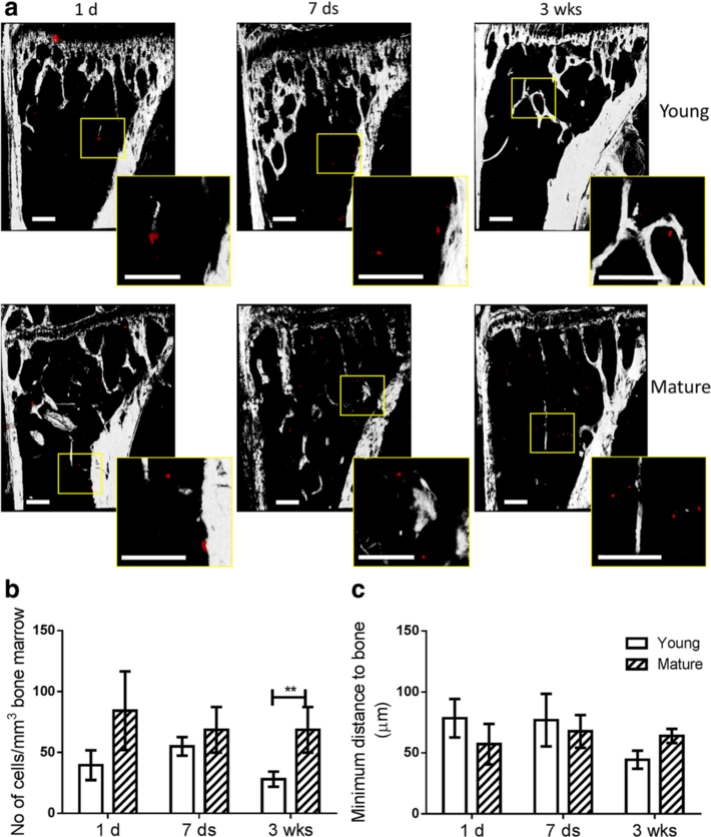
Quantification of human breast cancer cells arriving and colonizing the bone marrow of female mice. **a** The DiD labelled tumour cells entering the bone microenvironment were visualized and quantified using a two-photon microscopy on day 1, 7 and 21 post tumour injection. Scale bar = 200μm. **b** The number of tumour cells per mm^3^ bone marrow volume and **c** the minimum distance from tumour cells to bone surfaces were quantified and compared between 6- and 16-week old mice using the Volocity 3D Image Analysis software. *n* > 6, ** *P* < 0.01, t-test

To investigate whether different numbers of tumour cells seed to and colonize bone microenvironment of young and mature female mice, DiD labelled breast cancer cells were identified and quantified in tibial bone marrow at different time points (1 day, 7 days, and 3 weeks post injection) using two-photon microscopy *ex vivo* (Fig. 5a). There were no significant differences between young and mature mice at 1 and 7 days post inject, while significantly fewer tumour cells were found in young animals 3 weeks post injection (Fig. 5b). There was also no difference between younger and older female mice in the distances from tumour cells to the nearest bone surfaces (Fig. 5c).

### Bone turnover was higher in young compared to mature female mice

**Fig. 6:**
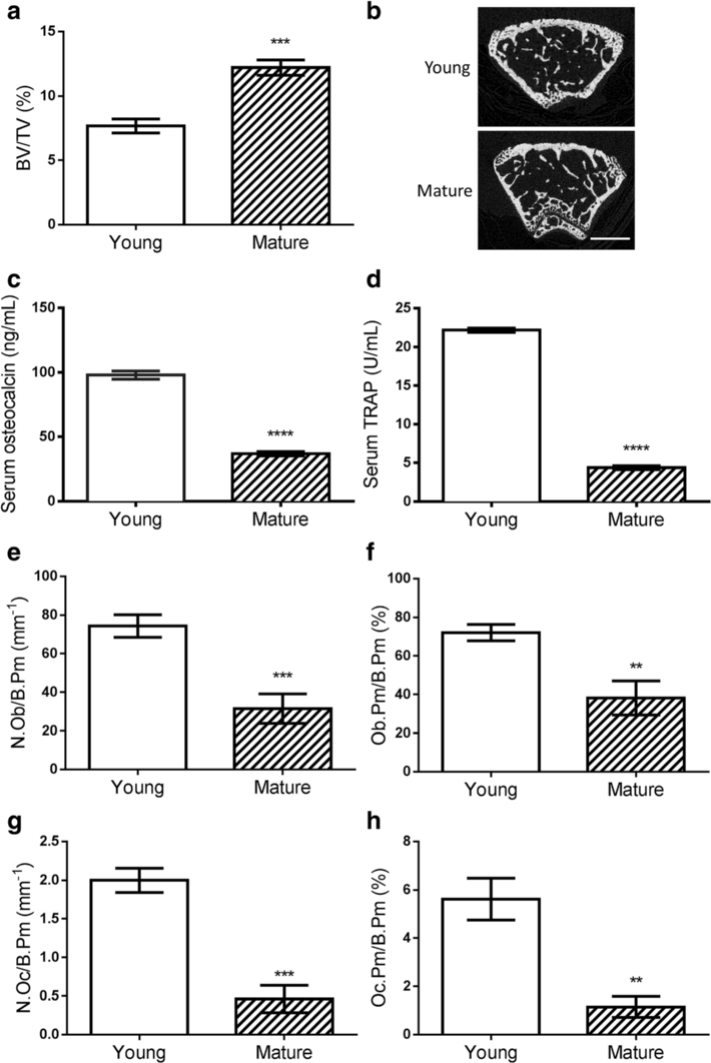
Differences of bone remodelling activities between young and mature female BALB/c nude mice. **a** & **b** BV/TV was compared between 6-week old (Young) or 16-week old (Mature) female mice using the micro-CT analysis. n > 6, scale bar = 500μm. **c** The bone formation marker (Osteocalcin) and **d** bone resorption marker (TRAP) were also measured in mouse serum sample by ELISA, *n* = 5. **e** The number of osteoblasts per mm endocortical bone surface (N. Ob/B.Pm), **f** bone surface covered by osteoblasts (Ob.Pm/B.Pm), **g** number of osteoclasts per mm endocortical bone surface (N.Oc/B.Pm), and **h** bone surface covered by osteoclasts (Oc.Pm/B.Pm) were all compared between young and mature mice. *n* > 4, * *P* < 0.05, ** *P* < 0.01, *** *P* < 0.001, **** *P* < 0.0001, t-test

To evaluate differences in bone remodelling activities, detailed analyses of bone structure were performed and phenotypes were compared between the young and mature female mice. Micro-CT analysis on femurs showed that the young female mice had 18% less BT/TV compared to mature animals (7.7 ± 0.5 vs 12.2 ± 0.6, *p* = 0.0007)(Fig. 6a and b). The young mice had evidence of higher bone formation (98.0 ± 3.2 vs 36.7 ± 6.7 ng/mL osteocalcin in serum samples, *p* < 0.0001)(Fig. 6c) and 4 fold greater bone resorption activity (22.2 ± 0.3 vs 4.4 ± 0.3 U/mL TRAP activity, *p* < 0.0001) than the mature mice (Fig. 6d). Quantified at cellular level and compared to mature mice, younger female mice have 136% more osteoblasts (74.4 ± 5.8 vs 31.5 ± 7.6 mm^−1^, *p* = 0.0007)(Fig. 6e) and 89% more osteoblast covered bone surfaces (72.1 ± 4.3 vs 38.2 ± 8.8 %, *p* = 0.0028)(Fig. 6f). In terms of osteoclasts, the young mice have 334% more osteoclasts (2.0 ± 0.2 vs 0.5 ± 0.2 mm^−1^, *p* = 0.0002)(Fig. 6g) and 389% more osteoclast covered bone surfaces (5.6 ± 0.9 vs 1.2 ± 0.4 %, *p* = 0.0018)(Fig. 6h).

## Discussion

The ‘Seeds and soils’ hypothesis suggests that both the metastasis initiating tumour cells and the microenvironment are equally important for the initiation of metastases [8]. To test this, we used different aged (6-week and 16-week old) immunocompromised mice as xenograft models to investigate the relationship between numbers of tumour cells colonizing bone, bone turnover, and the incidence of osteolytic skeletal metastases caused by prostate and breast cancer.

In the prostate cancer model, bioluminescent signals were clearly visible in the skeleton, three weeks after initial tumour cell injection. As bioluminescence detects bone marrow metastases of ≈ 0.5mm^3^ volume [27], the signals detected were indicative of overt, growing lesions. These lesions were associated with bone destruction that was confirmed by anatomical confirmation and micro-CT analysis post mortem. The results showed that both the frequency of skeletal metastases and tumour burden were higher in young mice compare to mature mice. However, there were no differences in the incidence of metastases in soft tissues. This indicates that the difference in the frequency of metastases across different aged cohorts is unique to the skeletal system and determined by the bone microenvironment. Further examination of the bone phenotype showed that young male mice had lower bone mass compared to mature animals but higher bone turnover. This was characterised by significant increases in the numbers of osteoblasts and osteoclasts, and elevated bone formation and resorption activities. This is consistent with clinical studies which suggested higher levels of bone turnover predict poor outcome in patients with bone metastasis [20, 22, 28]. To determine whether the higher metastasis frequency is due to higher numbers of metastasis initiating cells taking up residency in bone, we quantified the numbers of prostate cancer cells present in the bone marrow across a three-week period after initial injection, using two-photon microscopy and cell membrane labelling techniques. The results suggested that there were no increase in prostate cancer cells arriving in the bone marrow of young mice compared to mature animals and that these cells were in direct contact or one cell layer away from bone surface osteoblast lineage cells as they were within a range of 50 μm to the nearest bone surface on which an osteoblast has a typical diameter of 15-30 μm [29]. Surprisingly, there were significantly more tumour cells detected in the bone marrow of mature mice on day 1 and at 3 weeks post injection. This warrants further investigation. One explanation is that fewer DiD labelled tumour cells were detected in younger mice as a result of more proliferation and consequent loss of dye [24]. Another possibility is that the low bone turnover micro-environment in mature mice is more selective for stem cell-like cancer cells, while high bone turnover micro-environment in younger mice are more supportive to the growth of metastases and may correlated with angiogenesis which will be discussed in following sections. These data suggest that the higher frequency of skeletal metastases for prostate cancer in younger xenograft models is not due to increased tumour cells homing to the bone marrow but supports the hypothesis that it is the bone microenvironment that controls the frequency of growing metastases detected in these xenograft models, through induction of lesion initiation and growth [30, 31].

Similar results were found in the breast cancer model. Significantly higher frequencies and levels of tumour burden, determined by bioluminescence and anatomical confirmation post-mortem, were found in young female mice compared to mature mice. This was not due to higher number of breast cancer cells arriving into the bone marrow of younger animals, but correlated with the enhanced bone turnover activities confirmed by both ELISA of serum bone turnover marker (osteocalcin and TRAP) and histomorphometric analysis of osteoblasts and osteoclasts. Once again, there was a trend towards more tumour cells being detected in bone marrow of older female mice.

Taken together, the differences in frequency of skeletal metastases between young and mature mice in both prostate and breast cancer models strongly suggested that it is the trigger for the proliferation of resident tumour cells in bone that is linked to the differences in bone turnover in the bone microenvironment. This is consistent with previous findings of our own and of others that showed increased frequencies of bone metastases by prostate and breast cancer xenograft models in mature animals with experimentally induced enhancement of bone turnover [17, 18, 32]. More importantly, these data correlate with disease outcomes in cancer patients: Breast cancer patients presenting at a younger age (<40) have worse survival rates and more metastases compared to disease presented in older women, while younger men (<44) with high grade prostate cancer, have poorer prognosis compared to older men with a similar grade/stage distribution [33, 34]. One could argue these differences are due to higher available sex steroid hormone levels rather than the higher bone turnover in younger populations, directly affecting tumour growth. However, our data suggests another mechanism, as we intentionally used sex steroid hormone-independent cell lines: PC3 and MDA-MB-231 in these studies that are unaffected by androgens/oestrogens.

The xenograft model used in this study has limitations: firstly, we could not determine the role of the immune system, specifically T cells, in regulating metastatic frequency. The latter have been implicated in the bone metastatic process [35]. Secondly, the administration of a single high dose of cancer cells via intracardiac injection only partially mirrors seeding events in humans where low numbers of cancer cells are released into the circulation over prolonged time intervals. Finally, other differences between young and mature mice could also contribute to variations in metastatic frequencies such as the higher growth hormone levels and continuous longitudinal bone growth in younger mice. Particularly, differences in endothelial lineage populations and associated niches could also affect metastasis frequency and should be investigated [16]. Tumour cells were shown to be rapidly engrafted into bone marrow endothelial microdomains, where endothelial cells express higher level of adhesion molecules such as E-selectin, P-selectin, and intercellular adhesion molecule (ICAM-1) [36–38]. Kusumbe et al. recently showed that there are two distinct bone microvessels based on the expression of CD31 (also known as PECAM1) and Endomucin (Emcn), which were proposed as type H subset (CD31^hi^Emcn^hi^) and type L subset (CD31^lo^Emcn^lo^) [39]. The type H subset expresses higher level of metastasis related growth factor transcripts such as *Tgfb1* and *Tgfb3* [40]. The type H subset is a key to couple angiogenesis to osteogenesis and was strongly reduced in bone from aged animals [39]. This study suggested that the correlation of higher incidence of skeletal tumours in younger animals with the presence of type H microvessels in addition to higher bone turnover.

In conclusion, our study using two established metastatic cancer cell lines, provides further evidence to support the hypothesis that the frequency of overt skeletal metastases in hormone-independent prostate and breast cancer is determined by growth initiating influences within the bone microenvironment not by numbers of tumour cell initially seeding to these sites and provides further grounds for the use of treatments to suppress bone turnover and prevent development of skeletal metastases of prostate and breast cancer at early stages of disease.

## Abbreviations

BV/TV: Trabecular bone content
IVIS: In vivo imaging system
N.Ob/B.Pm: Number of osteoblasts
N.Oc/B.Pm: Number of osteoclasts
Ob.Pm/B.Pm: Bone surface covered by osteoblasts
Oc.Pm/B.Pm: Bone surface covered by osteoclasts
P1NP: Type 1 procollagen amino-terminal-propeptide
SRE: Skeletal-related events
TRAP: Tartrate-resistant acid phosphatase 5b
